# Tools for mass screening of G6PD deficiency: validation of the WST8/1-methoxy-PMS enzymatic assay in Uganda

**DOI:** 10.1186/1475-2875-12-210

**Published:** 2013-06-19

**Authors:** Mariana De Niz, Alice C Eziefula, Lucas Othieno, Edith Mbabazi, Damalie Nabukeera, Emmanuel Ssemmondo, Samuel Gonahasa, Patrick Tumwebaze, Deborah DiLiberto, Catherine Maiteki-Sebuguzi, Sarah G Staedke, Chris Drakeley

**Affiliations:** 1 Malaria Centre, Department of Infectious and Tropical Diseases, London School of Hygiene and Tropical Medicine, London, UK; 2 Infectious Disease Research Collaboration, Kampala, Uganda; 3 Current address: Malaria Group, Institute of Cell Biology, University of Bern, Bern, Switzerland

**Keywords:** Malaria, G6PD deficiency, WST8/1-methoxy PMS, Primaquine

## Abstract

**Background:**

The distribution of the enzymopathy glucose-6-phosphate dehydrogenase (G6PD) deficiency is linked to areas of high malaria endemicity due to its association with protection from disease. G6PD deficiency is also identified as the cause of severe haemolysis following administration of the anti-malarial drug primaquine and further use of this drug will likely require identification of G6PD deficiency on a population level. Current conventional methods for G6PD screening have various disadvantages for field use.

**Methods:**

The WST8/1-methoxy PMS method, recently adapted for field use, was validated using a gold standard enzymatic assay (R&D Diagnostics Ltd ®) in a study involving 235 children under five years of age, who were recruited by random selection from a cohort study in Tororo, Uganda. Blood spots were collected by finger-prick onto filter paper at routine visits, and G6PD activity was determined by both tests. Performance of the WST8/1-methoxy PMS test under various temperature, light, and storage conditions was evaluated.

**Results:**

The WST8/1-methoxy PMS assay was found to have 72% sensitivity and 98% specificity when compared to the commercial enzymatic assay and the AUC was 0.904, suggesting good agreement. Misclassifications were at borderline values of G6PD activity between mild and normal levels, or related to outlier haemoglobin values (<8.0 gHb/dl or >14 gHb/dl) associated with ongoing anaemia or recent haemolytic crises. Although severe G6PD deficiency was not found in the area, the test enabled identification of low G6PD activity. The assay was found to be highly robust for field use; showing less light sensitivity, good performance over a wide temperature range, and good capacity for medium-to-long term storage.

**Conclusions:**

The WST8/1-methoxy PMS assay was comparable to the currently used standard enzymatic test, and offers advantages in terms of cost, storage, portability and use in resource-limited settings. Such features make this test a potential key tool for deployment in the field for point of care assessment prior to primaquine administration in malaria-endemic areas. As with other G6PD tests, outlier haemoglobin levels may confound G6PD level estimation.

## Background

Malaria has exerted the greatest genetic pressure on the human genome in recent times, resulting in the evolutionary selection of genetic mutations that confer protection against the disease [1-4]. Glucose-6-phosphate dehydrogenase (G6PD) is an X-linked recessive hereditary disorder that currently affects 200–400 million people worldwide, with over 160 mutations identified [3-6] and there is pronounced geographical overlap between areas of G6PD deficiency prevalence and malaria endemicity [2,7-15]. The G6PD gene codes for an enzyme responsible for catalyzing nicotidamine adenine dinucleotide phosphate (NADP+) to its reduced form, NADPH, in the pentose phosphate pathway. Among G6PD variants with reduced enzyme activity, several phenotypic effects have been described, and are classified by the WHO as: enzyme deficiency with chronic non-spherocytic anaemia (class I, <10% activity); severe enzyme deficiency (class II, <10% activity); moderate/mild enzyme deficiency (class III, 10-60% activity); very mild or no enzyme deficiency (class IV, >60-100% activity); and increased enzyme activity (class V, >150% activity) [16]. Erythrocytes with insufficient G6PD are thus unprotected against oxidative injury, and individuals with G6PD deficiency may develop haemolytic anaemia in response to a number of stresses, including infection and exposure to medications such as the 8 amino-quinoline, primaquine [17].

Primaquine has received renewed interest in the context of malaria eradication. The drug is recommended as presumptive anti-relapse treatment of *Plasmodium vivax* and *Plasmodium ovale* infection due to its activity against hypnozoites. Furthermore, it remains the only readily-available drug that actively clears mature *P. falciparum* gametocytes [18-22]. Given the risk of haemolysis in G6PD deficient individuals, and the genetic and phenotypic variability of G6PD deficiency across geographic areas where primaquine treatment is considered, estimation of G6PD enzyme function prior to drug administration is recommended [23]. At present, however, primaquine therapy without prior determination of G6PD enzyme function, perhaps due to a lack of reliable tests, is thought to be common [24].

One possible reason for the current lack of a standard diagnostic test is that the majority of methods for assessing G6PD deficiency have shortcomings for field use in tropical countries [25-27] (see Table 1). In 2003, a novel enzymatic method to detect G6PD deficiency was developed [28], based on the WST8 tetrazolium salt and the 1-methoxy PMS hydrogen carrier. The assay has reduced light sensitivity, and is easily interpretable, both quantitatively and qualitatively. In 2010 Kuwahata *et al.* reported a version of this method, optimized for use in a 96-well plate format using dried bloodspots in filter paper, which was successfully tested as an in-field mass-screening tool for G6PD deficiency in the Solomon Islands [25]. The aims of this current study were to further validate the WST8/1-methoxy-PMS test by comparison with a commercially available enzymatic reference test and to assess the test’s robustness for field use.

**Table 1:** Available tests for determination of G6PD deficiency and their use in field settings

**Test**	**Characteristics**	**Shortcomings for field and mass-screening**
DNA sequence analysis of the G6PD gene.	Extremely reliable. Primers are used to check whether the G6PD gene contains a mutation.	Requires training, and equipment. Genotype does not correlate with enzyme function and the risk of haemolysis. Female heterozygous have unpredictable phenotype due to X chromosome lyonization. Only one mutation can be analysed with one primer (>160 mutations exist).
Brilliant cresyl blue decolouration test	Involves the action of G6PD and NADPH diaphorase. A deficiency of either one of these enzymes on RBCs would result in the brilliant cresyl blue remaining unchanged in the test.	Laborious processes; requires technical skill, and has low sensitivity.
Methaemoglobin reduction test	Based on the oxidation of Hb to MetHb by sodium nitrate and the subsequent enzymatic reconversion to Hb in the presence of methylene blue.	Laborious, qualitative and low sensitivity. Does not enable identification of heterozygous deficient females.
Formazan ring method	Uses the principle of the MTT-Linked spot test. When G6PD is present at normal levels, MTT is reduced to a purple insoluble formazan derivative, and results in a specific diameter of discolouration.	Prone to misdiagnosis.Ring thickness may be affected by exogenous factors.
Sephadex gel MTT-PMS method	Mostly used in Asia, and predecessor in concept, of the WST8/1-methoxy PMS test.	Reacts with haemoglobin; is light sensitive and water insoluble. It is of a qualitative nature.
Fluorescent spot test (FST)	ICSH-recommended method.	Its cut-off value for G6PD deficiency determination is only 10-20% of the normal G6PD activity, which excludes patients with moderate enzyme deficiency and increases the risk of false-normal diagnosis.
BinaxNOW® rapid test	Rapid test format: Overcomes issues of technical skill, sophisticated equipment and reliability.	It is highly dependent on temperature-sensitive kinetic enzymatic reactions. This limits its use to areas with temperatures between 18 and 25C. Potential cost.
CareStart™ test	RDT format. Qualitative chromatographic test, based in the reduction of colourless nitro blue tetrazolium dye to dark colour formazan. Long-term temperature stability.	Potential cost.
R&D® enzymatic test (reference)	Both depend on the conversion of NADP + to NADPH by G6PD. NADPH converts colourless tetrazolium salt into a coloured formazan, while NADP + does not.	Enzymatic gold standard. Requires various temperature-dependent incubations.
WST8/1-methoxy PMS test (test under validation)		Evaluated in this work. Advantages: no reaction with haemoglobin, lower light sensitivity.

## Methods

### Study site & sample selection

The study was conducted in seven sub-counties (Nagongera, Paya, Kirewa, Kisoko, Petta, Mulanda, and Rubongi) in Tororo district, an area with very high malaria transmission intensity in Uganda. In August-September 2010, the study area was mapped and a census survey carried out. Households within 2 km of a health facility were included in the sampling frame. Children under the age of five years were recruited from randomly selected households and were enrolled into a cohort study (Clinical Trials registration number NCT01024426) if they met the following inclusion criteria: 1) age < 5 years, 2) agreement of parents or guardians to provide informed consent, 3) no intention to move during the follow-up period. Clinical and laboratory evaluations were conducted at enrolment and repeated every six months over the period of follow-up. Blood samples collected from cohort study participants at follow-up visits conducted in July and August 2011 were used for the G6PD study.

### Laboratory procedures

Blood samples were collected by finger-prick, onto 3MM filter paper, and were dried at ambient temperature. Samples were then stored at room temperature in zip-lock bags containing silica desiccant beads, and assayed within 24-72 h. The remainder of the sample was stored for various time periods and temperature/illumination conditions for further evaluation. Additionally, haemoglobin values were obtained using a HaemoCue B analyser. In parallel, two sets of internal controls were generated to calibrate the assay. A commercial standard reagent of known G6PD activity (Trinity Biotech Normal Control) was used to create a panel of normal, moderate, and severe deficiency (100%, 30% and 10% activity respectively), as well as a no-enzyme control (0%). The second set of internal controls was generated from human blood from two volunteers with normal G6PD activity, and followed the procedure described for the field-adapted test [25]. Each set of controls were spotted onto 3MM filter paper (Whatman), and stored under the same conditions as the samples. Blood spots and controls were tested by both the optimized WST8/1-methoxy PMS assay, and by the commercially available standard R&D® test. Results were evaluated both visually and quantitatively.

### WST8/1-methoxy PMS assay

The principle of the WST8/1-methoxy PMS method depends on reducing hydrogen from NADPH converting WST8 to WST8-formazan in the presence of the hydrogen carrier 1-methoxy-PMS. This reaction yields a strong easily detectable orange colour, with colour intensity directly proportional to G6PD activity. After a 2 hr incubation at room temperature, samples with normal G6PD activity show strong orange colour, deficient samples show faint colour (moderate deficiency likely to represent heterozygotes) or no colour (severe deficiency & negative controls).

Two stock solutions were prepared: a working mix, and a control mix. The working mix contained 50 mM G6P (Roche), 4 mM NADP (Merck Pty Ltd), 1M Tris–HCl pH 7.2-7.5, and 100 mM MgCl2 (Sigma-Aldrich). The control mix contained all reagents in the concentrations described above, but lacked NADP and G6P. Mixes for assay development consisted of 0.5 mL of WST8/1-methoxy PMS (Dojindo Laboratories), 0.5 mL of working stock solution, and 19mL of distilled water for every 96-well plate. Negative controls were generated on site as described in previous studies [25]*.*

A 1.5 mm diameter disc was punched out from each blood spot sample and placed inside a single well of the 96-well flat bottom microplate. Samples were assessed in duplicate. Plates were incubated for 2 h at ambient temperature, and were then inspected by eye by two different observers for qualitative analysis. For quantitative analysis, the optical density was quantified in a microplate reader (Multiskan EX, Thermo scientific) at wavelength OD450-594 nm_._ G6PD levels were determined in reference to the control panels.

### Reference assay: standard quantitative G6PD assay (R&D® diagnostics)

The R&D® colourimetric test was used for validation [29,30]. In this test, the resulting NADPH reacts with a colour reagent in which a formazan salt (nitrotetrazolium blue) is produced, generating a visually detectable purple colour. The resulting OD (measured at 550 nm), is proportional to the level of G6PD present in the dried sample. The assay was performed using 96-well plates and dried blood-spots in filter paper as per manufacturers instructions. The same sets of controls were used for both assays, and their robustness tested under various temperature, storage and light conditions.

### Experiments to assess assay robustness

#### Storage

Storage of 150 dried blood spots (FPBS) prior to development of the assay was done at 24°C and 4°C and tested at days 1, 2, 4, 5, 9, and 10 post-collection. Working mixes were stored at 24, 4, and −20°C, and tested at weeks 1,2 and 3.

#### Reaction stability

Control assays were developed for 2 hrs at 3 different temperatures (37°C, 24°C and 10°C) to determine whether the kinetics of the assay was affected by temperature. Given the identification of limitations related to storage at room temperature of blood-spots in filter paper previously reported [25], a selection of samples were assayed 24 h after collection of the sample, and frozen at −20°C immediately after absorbance was quantified. They remained frozen for 1, 2, 3 and 4 weeks before G6PD assessment was carried out again both qualitatively and quantitatively.

#### Light

The 2 hr development of assays for G6PD determination was done under various light conditions: in the dark, scattered light (indoors), and direct exposure to sunlight (outdoors).

#### Filter paper use

Assays for filter-paper saturation with blood-spots were done for both the WST8/1-methoxy PMS assay and the standard test, to assess whether or not significant differences in saturation could affect G6PD level determination. Such assessment was done by perforating 5–6 blood spots with different levels of saturation from the filter paper, and comparing the final quantitative readout.

For all measurements, the same preliminary experiments to those carried out with the WST8/1-methoxy PMS assay were reproduced with the standard reference test.

### Sample size

The sample size was computed based on G6PD deficiency prevalence previously calculated by two independent studies in Kampala (16%) [31,32]. To validate the WST8/1-methoxy PMS method by comparison to the reference test with 80% power, the minimum number of samples calculated was 108. The final number of samples compared was 122, and a further 113 samples were evaluated by the WST8/1-methoxy PMS method alone.

### Data entry and statistical analysis

Data regarding clinical evaluations, and G6PD assay outcomes were double-entered and validated. Visual analysis was done independently by two observers. Agreement scores between observers, G6PD level visual determination, and quantitative data were produced, and analysed with STATA version 11 (STATA Corporation, College Station, TX). For analysis of the use of the two tests, a contingency table was produced and sensitivity, specificity, PPV and NPV were calculated. A Receiver Operating Characteristic (ROC) curve was calculated. Potential characteristics that could affect G6PD deficiency assessment including gender, age, haemoglobin levels, and prevalence of anaemia were tested by univariate and multivariate regression analysis. Agreement between observers regarding qualitative G6PD activity levels by the WST8 assay, and the R&D reference test was determined by calculating a weighted kappa (Kw) value. A *p-*value < 0.05 was considered as statistically significant.

### Ethics

Ethical approval to perform the G6PD assay validation was obtained from the London School of Hygiene and Tropical Medicine Ethics Committee (application no. 010/361). The use of human participant samples from the ACT PRIME study was under ethical approval of the Makerere University School of Medicine Research and Ethical Committee (no. 2010–108), the Ugandan National Council for Science and Technology (no. HS 794), the LSHTM Ethics Committee (no. 5779), and the University of California San Francisco (no. 006160).

## Results

### WST8/1-methoxy PMS test use in a field setting

#### Timeframe and temperature storage conditions affect assay performance

**Figure 1:**
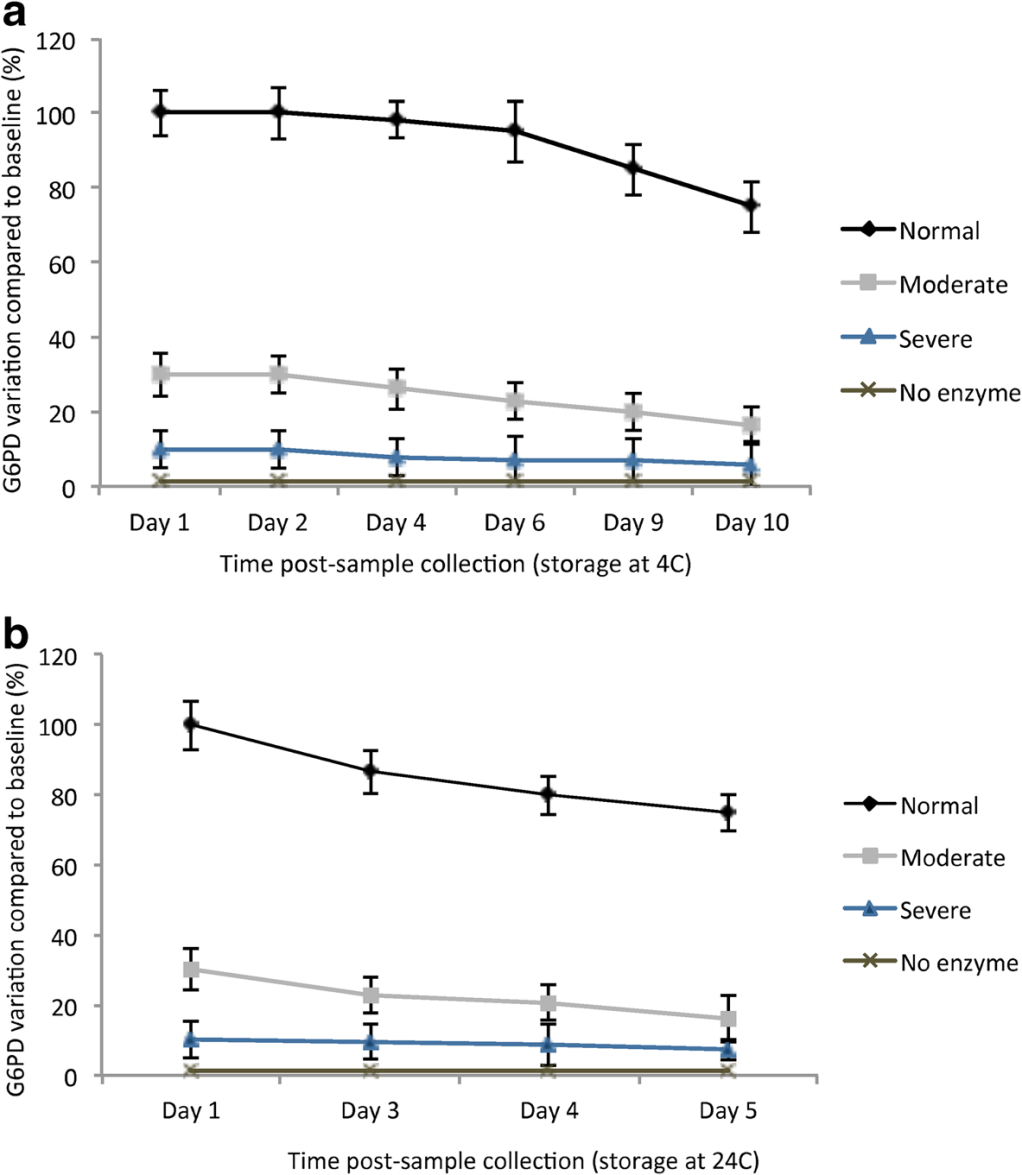
**Enzyme degradation due to storage on filter papers. a**) 150 filter papers with control blood spots with normal activity, moderate deficiency, severe deficiency, and no enzyme (100%, 30%, 10%, and 0%) were stored for up to 10 days at 4°C, and their activity measured at days 1,2,4,6,9, and 10. **b**) Samples were stored at room temperature, in the dark, for days 1–5, and the activity measured daily.

**Bloodspot storage** Following collection, a random selection of 150 FPBS were stored at two different temperatures (4°C and 24°C), and assayed at various days (1, 2, 4, 5, 6, 9, 10) to determine optimal storage times before degradation of G6PD occurs and risk of misclassification increases. G6PD enzymatic activity could still be accurately assessed 10 days after sample collection with storage of blood spots at 4°C. Beyond this timeframe, the risk of misclassification increased (Figure 1a). For samples stored at room temperature, enzyme degradation occurred at a faster rate than previously reported [25], and classification at day 5 post-sample collection was not possible due to a high degree of misclassification (Figure 1b).

##### Assay mix storage

The stability of assay concentrated mixes was evaluated for a three-week time frame following storage at room temperature, 4°C and −20°C. Assays were then developed and ODs measured at time 0, and weeks 1, 2, and 3. Results for assay mixes stored at room temperature and 4°C were comparable to those obtained by Kuwahata *et al.*[25]. Results for assay mixes stored at −20°C yielded comparable results to those obtained using fresh mixes at all time points evaluated.

##### Assayed plate storage post-development

**Figure 2:**
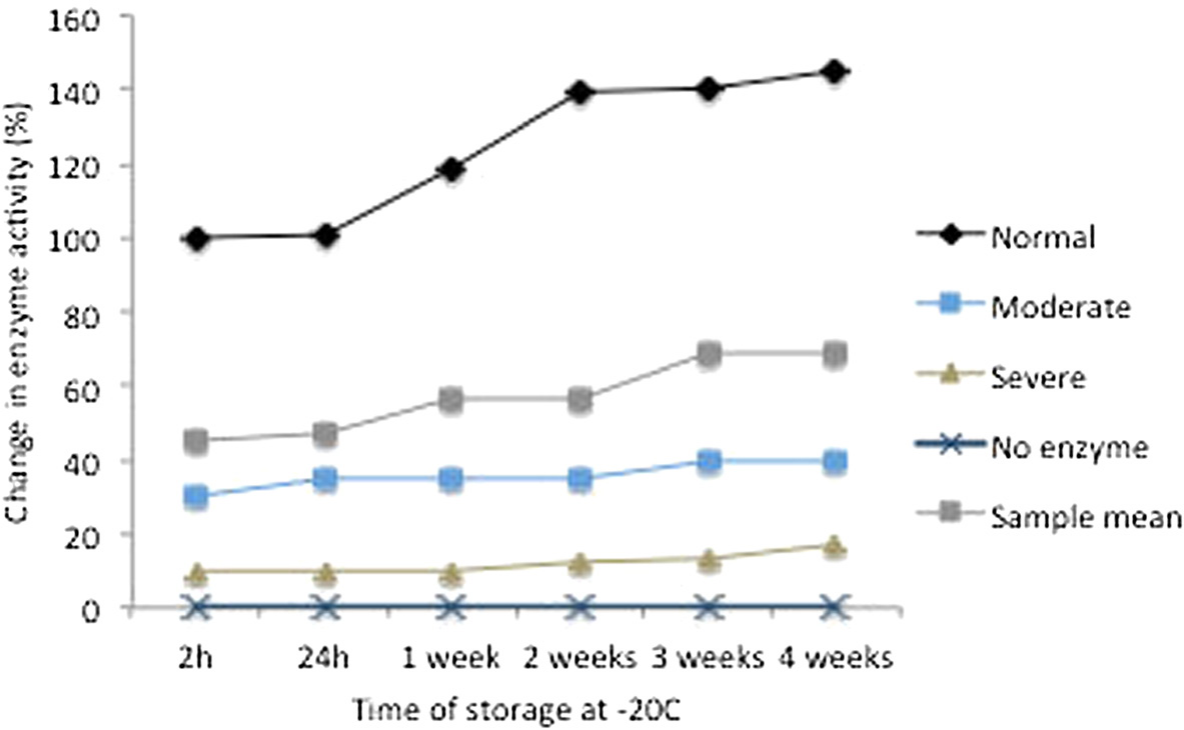
**Temperature effects on storage of developed assays.** G6PD activity was measured by the WST8/1-methoxy PMS test on fresh samples. The developed assay was then stored at −20°C for 24 h, and 1–4 weeks.

As the storage time of blood spots prior to assay was limited due to enzyme degradation, developed plates were re-assayed after initial assessment, after storage at −20°C for various time points including 24 hours, 1, 2, 3 and 4 weeks. Figure 2 shows that both visual and quantitative assessment of samples evaluated using the WST8 test was possible at all time points.

#### Temperature and scattered light had little effect on G6PD classification and assay performance

**Figure 3:**
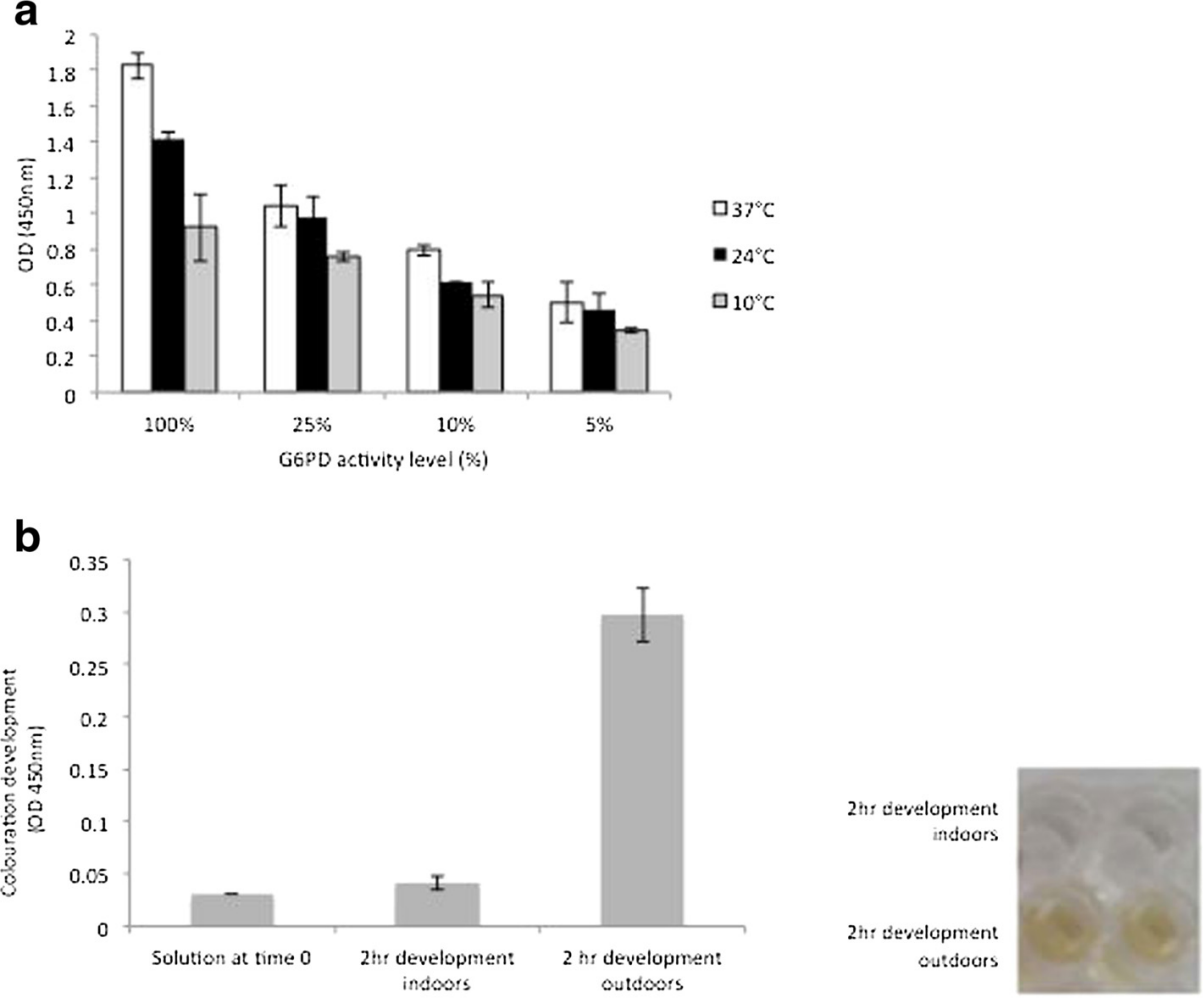
**Assay kinetics at various temperature and light levels. a**) G6PD activity measured after 2 hr development at 37°C, 24°C, and 10°C. Classification of G6PD values was possible at all temperatures, with 10°C showing the least variation Results repeated 3× in duplicate (p > 0.05). **b**) Colouration development in reagents only, following 2 hr incubation outdoors (exposure to sunlight), and indoors (exposure to scattered light). Aberrant colouration measured at the same wavelength as the G6PD assay (OD 450 nm) was detected.

Half-hourly kinetics of assays developed at 10, 24 and 37°C were measured and shown in Figure 3a. It was observed that G6PD level assessment and classification was not compromised across temperature ranges, although G6PD level assessment at 10°C was complicated (Figure 3a). In terms of assay sensitivity to aberrant colouration due to light, exposure of the assay to scattered light had little effect on abnormal colour development during a 2 hr period, however, direct exposure to UV light led to aberrant colour development (Figure 3b).

### Test validation by comparison to reference test

#### High inter-observer reliability exists for qualitative classification of G6PD levels using the WST8 test

A weighted kappa statistic (Kw) for inter-observer reliability (based on qualitative G6PD classification by two observers visual assessment) was calculated to be 0.922, indicating excellent agreement. Paired assessment was conducted for 122 samples. Most mismatches between observers occurred for samples with G6PD levels with threshold values between mild deficiency (30-60% activity) and normal activity. Such range of G6PD levels is not of significant clinical relevance. A 90% agreement between both observers and the quantitative estimation of enzyme activity was calculated, and 10% discordance in samples with borderline G6PD values (at the normal/moderately deficient threshold) was found. Importantly, moderate and severe deficiency values were always accurately classified. It was observed that fresh human blood controls led to an estimation of a significantly higher percentage of G6PD deficient samples (in the 10-20% activity range), than the commercial control (p < 0.05). Controls with similar storage time-frames as the samples being tested were used, in order to prevent misclassification due to higher or lower reference OD values, as has been also reported elsewhere [25,30,33].

#### The WST8/1-methoxy-PMS test has high agreement with the reference test

**Figure 4:**
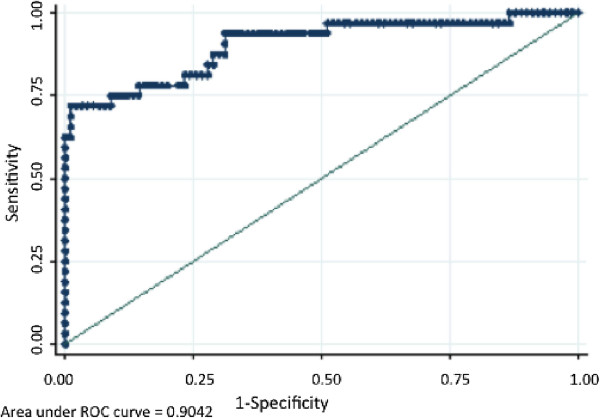
**Validation of the WST8/1-methoxy PMS assay (AUC).** Receiver operating characteristic curve for the performance of the WST8/1-methoxy PMS test for G6PD diagnosis in the field study in Uganda.

Agreement values between the WST8/1-methoxy-PMS assay and the reference R&D test were assessed using the categorization of G6PD enzyme function into a) severe deficiency (<10% G6PD activity), moderate deficiency (10-30%), mild deficiency (30-60%), and normal activity (60-100%). Results are shown in Table 2. There was 100% agreement in classification of severe, moderate, and >150% activity samples. The lowest agreement recorded occurred near the cut-off point between normal and mild deficiency values (40-60% enzyme activity). Importantly, both the WST8 test, and the R&D test enabled identification of individuals with low G6PD enzyme activity with the highest risk for haemolytic anaemia (<30% activity). Using the R&D test as a reference standard, the WST8 test’s overall sensitivity for G6PD normal or G6PD deficient was found to be 72%, specificity 98%, PPV 91.3%, NPV 91.9%. The overall percentage of correct diagnosis was 91.8%, and an AUC value of 0.904 was calculated (Figure 4).

**Table 2:** Detection of G6PD deficiency levels: agreement and validation of WST8/1-methoxy PMS test

	**WST8/1-methoxy PMS**	**Standard colourimetric test**	**Agreement (%)**
Total samples tested	122	122	-
Normal activity	98 (80.4%)	94 (77.04%)	92.63%
Mild deficiency (30-60% activity)	15 (12.3%)	21 (16.4%)	96.72%
Moderate deficiency (10-30% activity)	9 (7.38%)	9 (7.38%)	100%
Severe deficiency (<10%)	0 (0%)	0 (0%)	100%

#### Study population and assessment of G6PD enzymatic activity

**Figure 5:**
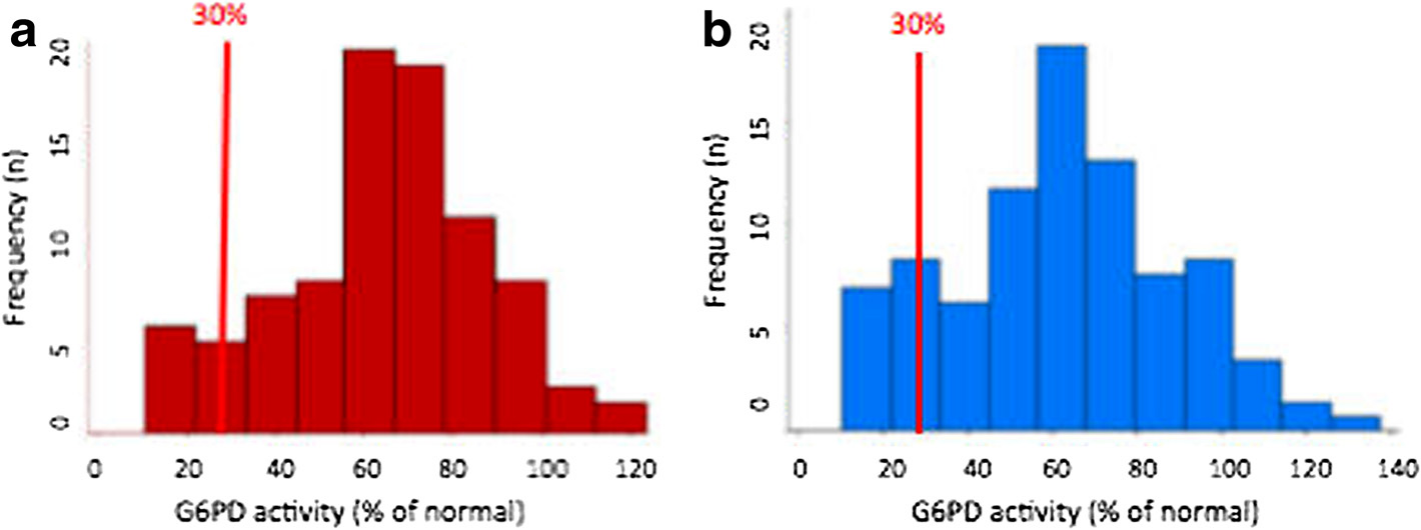
**G6PD distribution by gender. a**) Among 110 females, 84.5% had G6PD levels ranging from 60% to 123% activity; 9.37% of females had G6PD activity lower than 30% - the activity threshold established by the WHO as posing a risk for primaquine administration at the present regime. Most females had activity values between 60 and 80%. **b**) Among 125 males, 76.8% had G6PD levels ranging from 40.3% to 137.8%. 9.4% of males had values lower than 30% activity. Most males had activity values in the 60-70% range.

Samples from 235 children (110 females, 125 males) were analysed. No significant difference in G6PD deficiency levels between males and females was found (p = 0.136) by either the WST test or the reference R&D test. Among the male children, 16.5% showed intermediate levels of G6PD activity (Figure 5). Mean age and age distribution was similar among all G6PD classes (normal, mild, moderate and low deficiency) (p = 0.802). No severe deficiency was detected in this study population. While children with severe G6PD deficiency were not seen, G6PD values as low as 10.3% activity were identified. Anaemia prevalence (defined in this case as Hb levels under 10g/dl) was not significantly different between G6PD classes (p = 0.072). However, overall haemoglobin levels between the 3 main G6PD classes was significantly different (p = 0.022), with general haemoglobin levels being lower in G6PD normal children (Table 3).

**Table 3:** Baseline measurements and G6PD classification by the WST8/1–methoxy PMS test

	**Glucose–6–phosphate dehydrogenase classification**				
	**Normal (40–100 + % act.)**	**Moderate (mild: 20–40% activity)**	**Moderate (low: 10–20% activity)**	**Summary statistic (univariate normal/deficient)**	**Total**
Number					235
Males (n)	96	21	8	p = 0.136	125 (53.2%)
Females (n)	93	10	7		110 (46.8%)
Age (years, range)	2.83 (0.1–5.3)	2.89 (0.01–5.8)	2.87 (0.01 –5.1)	p = 0.802	2.84 (0.01 – 5.8)
Hb level (g/dl, mean, range)	11.18 (4.9–14.9)	11.74 (8–16)	11.90 (8.8 – 17)	p = 0.022	11.3 (4.9 – 17.0)
Anaemia (%)	28.57	16.12	20.0	p = 0.072	26.4%
G6PD activity (%, range)	72.6 (40.3–137.8)	29.2 (20.5–39.6)	15.11(10.3–19.8)	n/a	63.2 (10.3–137.8)

## Discussion

Susceptibility of G6PD deficient individuals to haemolysis caused by anti-malarial drugs such as primaquine and other 8-aminoquinolines is a concern for worldwide efforts for malaria eradication, given the geographical overlap between malaria-endemic areas and those populations with high prevalence of G6PD deficiency [12,13,27]. While primaquine administration without G6PD screening for confirmed malaria cases is thought to be relatively common, ethical issues regarding the use of the drug are regaining attention as wider community use is considered. At present, a main limitation for wide-scale implementation of G6PD screening is the lack of a robust, low-cost and rapid test that can accurately classify the majority of samples obtained from individuals in a steady state (ie. not suffering from haemolytic anaemia at the time of test), and that enables testing of a large number of samples simultaneously. In 2003, Tantular and Kawamoto published a simple screening method for detection of G6PD deficiency based on enzymatic activity, with improved performance and reagent stability compared to its predecessors [28]. Additionally, the method offers the advantage of enabling both qualitative and quantitative assessment of G6PD levels based on the NADPH concentration in the test, which yields strong colouration. Since its description in 2003, the assay has been used in various settings, including Thailand [12] and Suriname [26]. In 2010, Kuwahata *et al.* successfully optimized the WST8/1-methoxy PMS assay for field use by adapting it to a 96-well plate format, and dried blood-spots in filter paper. The optimized test was successfully used to determine G6PD deficiency prevalence in Isabel Province, Solomon Islands [25]. Given the observations previously described regarding the performance of the WST8 test, the aims of this study were to validate the WST8 method in relation to a standard reference enzymatic test (commercially available R&D); and to identify operational shortcomings and advantages of this test for use in field and resource-limited settings.

The assay was found to be easy to use, with low use of consumables, and low requirements for sophisticated equipment, as well as being less time consuming than the reference test. On average, processing time for a 96-well plate worth of samples took 10 minutes of active processing and a 2 hr waiting period for development, while the R&D test took 1 hour of active processing. The WST8 test was not overly affected by temperature variation, and the temperature range within which accurate G6PD classification was possible, includes temperatures generally observed in tropical areas. Similarly, the test was less sensitive to scattered light in the laboratory than previously reported for other tests. Nevertheless, from our observations, we suggest avoiding unnecessary exposure of the test and the reagents to light for extended periods. In terms of storage of assay mixes and reagents, our conclusions are similar to those previously reached by Kuwahata *et al.* and confirm that long-term storage is advantageous for assay transport and assay use in field settings where assays may need to be run in the field, and subsequently tested in a central laboratory. This is likely, as a major limitation for storage is that enzyme degradation occurs in blood spots in filter papers limiting the time they can be held prior to testing. Previously, Kuwahata *et al.* determined that accurate G6PD classification could be done by the WST8 method on filter papers stored at ambient temperature for no more than five days, or alternatively at 4°C for up to 10 days. This is similar to the findings of this study for storage of samples at 4°C, yet storage of samples at room temperature for more than four days led to G6PD level misclassification. It is thus recommended that the samples be tested within 48-72h following sample collection, given that degradation time may vary slightly among different settings after this time frame. An alternative is the possibility to freeze assayed plates at −20°C for quantitation at a later time point. Although this requires freezing facilities, it would allow subsequent mass testing of samples for confirmation of visual readings. A key observation from this and previous studies [30], is that a control panel, which comprises positive controls and various levels of relative G6PD concentrations, should be stored in similar conditions to those of samples to be tested, as this will reduce the risk of misclassification. Two other observations, common to spectrophotometric assessments with FPBS were that both blood spot saturation and bubbles in the microplate wells can adversely affect reactions leading to aberrant readings and underestimation of G6PD levels. Overall, it was found that the WST8 assay offered major advantages in relation to other currently-used G6PD screening tests in its suitability for field use.

Importantly the assay also performed well. In comparison with the standard reference test, the WST8 test had 72% sensitivity, 98% specificity, and an AUC value of 0.904. The sensitivity of the test was only with misclassifications corresponding to samples with values between normal enzyme activity and mild G6PD deficiency i.e. individuals not at risk of severe haemolytic anaemia after treatment with primaquine (ie. >30% enzyme activity as defined by the WHO). Current tests, including the ICSH recommended fluorescent spot test (FST) method, report a sensitivity value as low as 32% [27,34-36]. In this context, the WST8 test enabled accurate identification of a wide range of G6PD enzyme levels. The study also showed a good inter-observer reliability (qualitative assessment) with very good agreement in relation to the quantitative classification though numbers of observers and samples were relatively few.

A known confounder for G6PD tests is haemoglobin concentration. This may be potentially attributed to the fact that in patients with haemolytic anaemia, older erythrocytes are haemolysed, while the remaining reticulocytes have normal or near-normal enzyme activity. Previous G6PD screening studies have therefore suggested that G6PD testing must be done in parallel with haemoglobin measurements [25,33], or that inbuilt haemoglobin normalization must be considered for accurate determination of status [30]. In this study, baseline haemoglobin measurements in G6PD normal and G6PD-deficient children were significantly different by univariate analysis. The lower haemoglobin levels in children with normal G6PD in this study may be attributable to G6PD deficiency being associated with a protective effect against infections that may result in anaemia, however, the study was not powered to test this effect. Importantly, no severe deficiency was detected in this study population using both the reference and WST8 tests, which is in agreement with the expected G6PD A- prevalent genotype in Africa [5,8].

In conclusion, the WST8 test offers some important advantages in comparison to other tests for G6PD deficiency assessment in large-scale screening studies and public health interventions where primaquine administration is being considered. As demonstrated by this study, G6PD screening using the WST8 assay can be easily nested into other public health interventions, which is advantageous for its inclusion in malaria elimination programmes contemplating the use of primaquine. Additionally, the high comparability of quantitative and qualitative G6PD estimates between the WST8- and the standard colorimetric tests used for diagnosis in hospital settings, suggest that the WST8 test would be a relatively safe basis for clinical decisions. This would obviously depend on existing clinical and laboratory capacity in any facility and require some adaptation to single sample testing [37]. No individuals with severe deficiency were identified in this study. Although this is a limitation in terms of validation of the test, a previous study carried out in the Solomon Islands with the WST8 test [25], enabled the identification of severely G6PD deficient individuals, as well as a range of G6PD activities similar to the one reported here. In order to fully assess the capacity of the test in various field settings, further studies in various geographical locations where diverse G6PD genotypes are prevalent, would be advantageous. A further key observation is the need for parallel haemoglobin determination, emphasized in previous G6PD deficiency assessments [25,26,30,33]. This is likely to be of general benefit both in assessing the interpretability of the test and also may indicate other causes of anaemia and any required treatment. Overall, the WST8 test has a considerable potential as a diagnostic tool prior to primaquine administration in malaria-endemic areas as a point of care test and/or as a screening tool for assessing G6PD prevalence in large-scale screening studies in areas contemplating primaquine deployment.

## Abbreviations

FPBS: Filter paper blood spots; FST: Fluorescent spot test; G6P: Glucose-6-phosphate; G6PD: Glucose-6-phosphate dehydrogenase; ICSH: International Council for Standardization of Haematology; NADP: Nicotinamide adenosine dinucleotide phosphate; NADPH: Nicotinamide adenosine dinucleotide phosphate reduced; WHO: World Health Organization; WST8/1-methoxy PMS: 2-(2-methoxy-4-nitrophenyl)-3-(4-nitrophenyl)-5-(2,4-disulfophenyl)-2H tetrazolium monosodium salt/ 1-methoxyphenazine methosulfate.

## Competing interests

The authors declare that they have no competing interests.

## Authors’ contributions

MDN, ACE, SS, CD wrote and edited the manuscript. MDN carried out the G6PD assay experiments and testing. MDN, ACE, CD carried out data analysis. CMS, DD, SG, PT and SS facilitated and supported the survey. MDN, EM, DN, LO, ES carried out sample collection and/or clinical assessment of the children in the study cohort. CD, ACE, and SS conceived the study. CD, ACE, SS, and MDN contributed to experiment design. ACE, SS, CD were involved in supervised field coordination. All authors read and approved the final manuscript.
